# TIMA SLAM: Tracking Independently and Mapping Altogether for an Uncalibrated Multi-Camera System

**DOI:** 10.3390/s21020409

**Published:** 2021-01-08

**Authors:** Omer Faruk Ince, Jun-Sik Kim

**Affiliations:** Center for Intelligent and Interactive Robotics, Korea Institute of Science and Technology, Seoul 02792, Korea; 023967@kist.re.kr

**Keywords:** SLAM, multi-camera, self extrinsic camera calibration, multi-edge based optimization

## Abstract

We present a novel simultaneous localization and mapping (SLAM) system that extends the state-of-the-art ORB-SLAM2 for multi-camera usage without precalibration. In this system, each camera is tracked independently on a shared map, and the extrinsic parameters of each camera in the fixed multi-camera system are estimated online up to a scalar ambiguity (for RGB cameras). Thus, the laborious precalibration of extrinsic parameters between cameras becomes needless. By optimizing the map, the keyframe poses, and the relative poses of the multi-camera system simultaneously, observations from the multiple cameras are utilized robustly, and the accuracy of the shared map is improved. The system is not only compatible with RGB sensors but also works on RGB-D cameras. For RGB cameras, the performance of the system tested on the well-known EuRoC/ASL and KITTI datasets that are in the stereo configuration for indoor and outdoor environments, respectively, as well as our dataset that consists of three cameras with small overlapping regions. For the RGB-D tests, we created a dataset that consists of two cameras for an indoor environment. The experimental results showed that the proposed method successfully provides an accurate multi-camera SLAM system without precalibration of the multi-cameras.

## 1. Introduction

In robotics, vision-based simultaneous localization and mapping (SLAM) is a geometric problem of mapping an unknown environment while tracking the camera pose simultaneously. Vision-based SLAM systems have a wide range of applications, such as autonomous navigation, search and rescue in natural disasters, and undersea exploration. Thus, the most important characteristics required in the SLAM system are to be accurate and robust. Liu et al. [1] show that a camera with a wide field of view provides more accurate camera localization and more robust mapping. In [2], it is also explained the advantages of a wide field of view in the problem of place recognition and geometric SLAM.

One of the solutions to obtain a wide field of view is to use a single camera with an omnidirectional lens. Yet, in [3], it shows that images taken with an omnidirectional lens suffer from wide image scale variation and low angular resolution. Thus, a pixel measurement of an omnidirectional camera is less accurate than that of a perspective camera. Another solution for enlarging the field of view is using multiple cameras. Approaches in [4,5] noted that using multi-cameras has many advantages in terms of providing more efficient localization and higher error tolerance compared to single camera usage. As in our previous study [6], we aim to use multiple cameras in this paper.

The primary challenge to be discussed in the use of multi-camera SLAM is the calibration of extrinsic parameters between cameras. For efficient localization and mapping, a SLAM system requires the integration of each information from cameras. For this reason, the available methods are reviewed depending on the necessity of multi-camera calibration.

SLAM systems where all cameras work as a single unit require precalibrated extrinsic parameters. The first example of this approach is the multi-camera parallel tracking and mapping with a nonoverlapping field of view (MCPTAM) [7], which is the multi-camera version of PTAM. To prevent tracking loss as much as possible, the authors used a large field of view cameras (omnidirectional). Thus, the camera model has been replaced by a polynomial model, and this leads to making some changes such as the epipolar correspondence search. Another example that requires precalibrated extrinsic parameters is Multicol-SLAM [8]. Multicol-SLAM extended the ORB-SLAM to a multiple fisheye camera usages and modified some pipelines of the ORB-SLAM in several aspects. First, they introduced a multi-keyframe concept. Second, the authors proposed the hypergraph formulas in an optimization process. Lastly, a loop closure thread revised in the multi-camera scenario is also one of their contributions. One of the recent examples of a multi-camera SLAM system is proposed by Yang et al. [9]. The authors draw attention to the difficulties of offroad navigation for SLAM systems and develop a new vision-based SLAM system. Their main contributions are listed as follows: building a spatial sensing model of a multi-camera system considering the system’s imaging principle, designing a multi-camera collaborative SLAM framework using the advantages of a large field of view for recovering the scale, and using deep learning for detection of repetitive scenes that provides more accurate loop detection. Lately, Kuo et al. also proposed another method of a multi-camera SLAM system that requires precalibrated extrinsic parameters [10]. The authors aim to develop a SLAM system that is compatible with arbitrary multi-camera configurations automatically. For this purpose, they come up with three main contributions that are an adaptive initialization system, a sensor-agnostic, an information-theoretic keyframe selection method, and a scalable voxel-based map management approach.

On the other hand, some approaches do not require the extrinsic parameters between cameras in advance. For instance, Heng et al. [5] developed a self-calibrated SLAM system with four cameras mounted on a micro aerial vehicle (MAV). On the MAV, they set the back and the front camera pairs up according to the stereo configuration. For online self-calibration, at least one stereo camera must be calibrated in advance. To recover the relative motion of the MAV, the authors proposed the minimal and linear 3-point algorithm that uses the information from the 3-axis gyroscope. Another self-calibrated multi-camera slam system is proposed by Carrera et al. [11]. In this approach, it is not necessary to have an overlapping region between cameras. However, there is a requirement that the robot must rotate the spot 360 degrees to ensure that all cameras see the same features. Each camera is localized according to the other cameras in the system. Then, the extracted maps from each camera are merged using 3D alignment, and all the map components are optimized. Still, the requirement of the robot turning 360 degrees is a constraint, and this could be considered as a precalibration method. A recent example of an autocalibration method for a multi-camera system is proposed by Feng et al. [12]. Their approach is calibrating a multi-camera system without any overlapping regions using SLAM. For this purpose, they first reconstruct the 3D model of the environment using SLAM and calibrate each camera through 2D-3D correspondences between the image sequences and the extracted 3D model.

**Figure 1:**
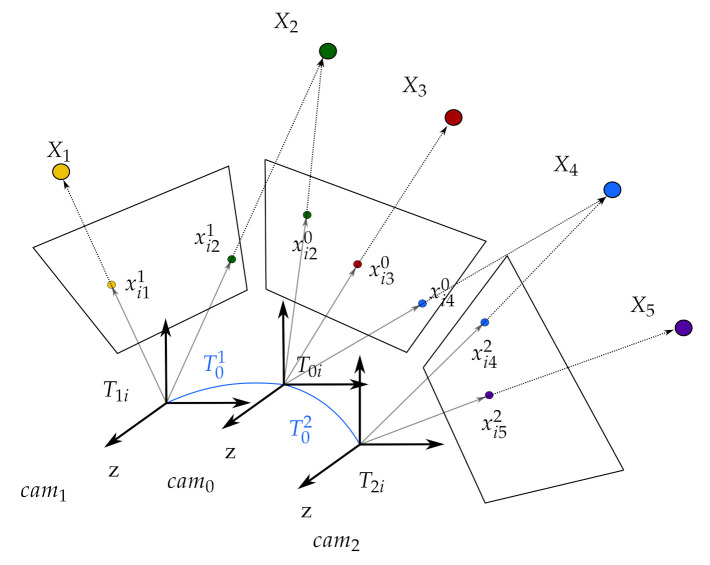
A multi-camera simultaneous localization and mapping (SLAM) system. The extrinsic parameters between cameras are indicated in blue. Observing the same points with cameras looking from different viewpoints provides a great advantage for robustness.

Whether a multi-camera SLAM system precalibrates or calculates the extrinsic parameters online, slight errors in extrinsic parameters may cause significant errors in the reprojection of map points, and this causes failure during initialization and tracking. To overcome this issue, we present a novel solution to an uncalibrated multi-camera SLAM system. We aim to develop a robust multi-camera SLAM system in which each camera is tracked independently on a single map, and the extrinsic parameters are estimated online. In the camera tracking, we do not use the extrinsic parameters explicitly to avoid the tracking loss by the error in the parameters which affect robustness. However, in the mapping stage, we still estimate and refine the extrinsic parameters to gather and utilize all the observations from the multiple cameras that improve the map accuracy. Figure 1 presents an example of a multi-camera rig.

Like the monocular SLAM systems, our system has a scale ambiguity problem for RGB cameras. RGB cameras are not able to set the scale factor (length of translational movement) concerning corresponding feature matching. Even though the proposed system uses multiple cameras, it is not possible to solve the scale ambiguity since the extrinsic parameter calculation is not in the Euclidean space. For resolving the scale ambiguity problem, RGB-D camera usage is one solution. RGB-D sensors provide both the Euclidean geometry and the image texture of an environment by integrating a depth map measured by a depth sensor, and color information comes from an RGB camera. We resolve the scale ambiguity problem since a depth sensor presents the scale information, and the proposed system is compatible with RGB-D cameras.

Since each camera performs its tracking, the proposed method might be considered similar to the collaborative SLAM systems. However, there are some significant differences. In collaborative SLAM systems like [13,14], multiple agents create individual maps, and a server merges those maps for a global map. Each camera runs independent processes such as tracking, local mapping and loop closing. Compared to this, the proposed method has a single shared map for all the cameras, while the cameras are tracked independently on the same map. In addition, there are single local mapping and single loop closing threads for all the cameras by utilizing the fact that all the cameras are fixed in the whole sequence.

## 2. Framework of the Proposed the Multi-Camera SLAM System

**Figure 2:**
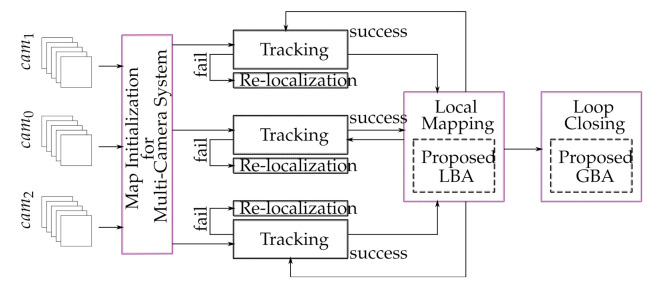
Block diagram of the proposed multi-camera SLAM system.

Our method extends the state-of-the-art monocular ORB-SLAM2 [15] algorithm to an uncalibrated multi-camera SLAM system. Figure 2 represents the block diagram of the proposed multi-camera SLAM system, and our contributions are in the blocks in purple. The main contributions that make the proposed method different from conventional multi-camera SLAM systems can be summarized as follows:

- SLAM framework for an uncalibrated multi-camera system in which each camera is tracked independently on a shared map.
- Online calibration of extrinsic parameters in the multi-camera system
- Initialization of multi-camera configuration.
- Simultaneous refinement of the SLAM components and extrinsic parameters by a multi-edge based graph optimization.

The SLAM components are described in the Table 1 and illustrated in Figure 1 for a better understanding of the proposed method.

### 2.1. TIMA SLAM

In conventional multi-camera SLAM systems, all cameras work as a single unit. To work as a single unit, such systems use given extrinsic parameters between cameras from the first frame to the last frame. In cases where the extrinsic parameters are not accurate, the system may fail on the map initialization or camera tracking due to the wrong map point projection from multiple cameras. As a solution to this problem, our method introduces two new concepts. The first one is an uncalibrated multi-camera system in which each camera is tracked independently on a single map. The second one is self-calibration and refinement of the extrinsic parameters for better localization and mapping.

When each camera works independently, the system does not need to use precalculated extrinsic parameters, which could be inaccurate. Unlike conventional multi-camera SLAM systems, the proposed independent tracking of each camera is not affected by the incorrect extrinsic parameters, even though they observe the same map points. Furthermore, in case a multi-camera SLAM system that operates as a single unit loses its track, it cannot relocalize itself unless it revisits the places previously observed. However, in the proposed system, as long as just one camera keeps tracking, the system can relocalize the other cameras by obtaining the reference keyframe tracking camera.

In the mapping process, we collect observations from all the cameras and estimate the map points and keyframe poses. We also estimate the extrinsic parameters simultaneously for more accurate mapping. Once the system calculates the initial extrinsic parameters online, it updates them iteratively to estimate a more accurate map. The optimization process will be explained in detail in Section 2.5.

Considering the advantages of the proposed method, we call it *‘TIMA SLAM–Tracking Independent and Mapping Altogether for an uncalibrated multi-camera system’*. This is because all the cameras in the system are being tracked independently while a map is built by observations from all cameras.

### 2.2. Multi-Camera Initialization

In the TIMA SLAM, despite operating multiple cameras, the initial map is generated by a camera selected as a reference. For that purpose, the system calculates the relative poses between the consecutive frames and triangulates the map points. For the relative pose estimation, two geometric models are evaluated in parallel. According to the assumption of planar and nonplanar scenes, the system calculates a homography and the fundamental matrix respectively. The selection of the appropriate model is determined by the equation given in [15]. Additionally, the system rearranges the associated motion hypotheses. At last, the system performs the global bundle adjustment (GBA) for the initial map optimization.

After the reference camera generates the initial map, each nonreference camera in the system localizes itself using the reference keyframe and the map points sent by the reference camera. For the nonreference camera localization, the system estimates each camera pose by performing RANSAC iterations with the perspective-n-point(PNP) algorithm [16]. If enough inliers support the pose of a camera, the system optimizes the pose and searches for more matches. Lastly, if there are still enough inliers, the system performs one more camera pose optimization and registers the camera. The same process applies to relocalize any camera in case it loses its tracking.

In the next stage, the extrinsic parameters (the relative poses between the cameras and the reference camera) are calculated upon the moment each camera localizes itself because of all cameras in the system being fixed to each other. Let $cam_{1}$ and $cam_{2}$ be the coordinate systems of the cameras and let $T_{cam1}^{W}$ and $T_{cam2}^{W}$ be their respective poses in world coordinate system W:

(1) $$T_{cam_{1}}^{W}=\left[\begin{array}{cc} R_{1} & t_{1}\\ 0 & 1 \end{array}\right]\;\mathrm{and}\;T_{cam_{2}}^{W}=\left[\begin{array}{cc} R_{2} & t_{2}\\ 0 & 1 \end{array}\right]$$

where $R_{1}$ and $R_{2}$ are the rotation matrices, and $t_{1}$ and $t_{2}$ are the translation vectors for $cam_{1}$ and $cam_{2}$ respectively. The relative pose between $cam_{1}$ and $cam_{2}$ is denoted as following:

(2) $$T_{cam_{1}}^{cam_{2}}=\left[\begin{array}{cc} R_{2}^{⊤}R_{1} & R_{2}^{⊤}\left(t_{1}-t_{2}\right)\\ 0 & 1 \end{array}\right]$$

**Figure 3:**
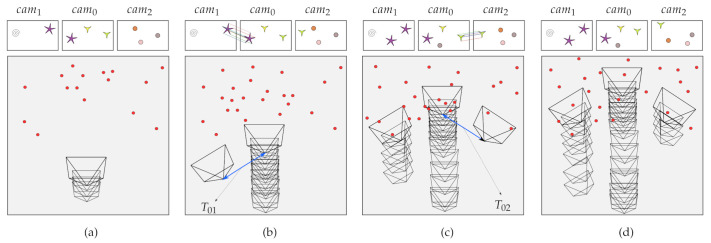
Multi-camera initialization of the system. Each subfigure consists of a big box which represents the sparse map and three small boxes which represent the current frames of corresponding cameras. The sparse map includes the keyframes (pyramids) and the map points (red). (**a**) First, the map is initialized with the reference camera. (**b**,**c**) In order to localize other cameras, we search for ORB correspondences between the reference keyframe and the current frame. Finally, we localize the other cameras and calculate the relative poses (blue). (**d**) Multi-camera initialization is completed.

Figure 3 presents the whole process of the multi-camera initialization with the initial estimation of extrinsic parameters. In this figure, the scenario shows the camera rig goes straight and the nonreference cameras are registered respectively. The big boxes represent the sparse map of the SLAM at different timestamps. Each big-box include three small boxes that illustrate the current frames of the corresponding cameras. The map on each timestamp shows the camera trajectory with created keyframes (pyramids) and the map points (red circles). For the localization of nonreference cameras, we look for ORB correspondences between the reference keyframe and the current keyframe of the nonreference camera. As soon as the nonreference camera completes the localization, we calculate the relative poses between the reference and the nonreference camera (blue lines) and complete the multi-camera initialization process.

### 2.3. Camera Tracking

Since each camera performs independent tracking, we use the original ORB-SLAM2’s tracking thread for the proposed system [15]. The tracking thread estimates the camera pose and makes a decision when to add a new keyframe. The system extracts FAST corners using the construction of a pyramid. A pyramid represents a group of the same signal or image at different scales, containing upsampling and downsampling by a constant scale factor. The tracking thread uses 8 scale level between pyramid levels with the scale factor 1.2. The extracted FAST corners are then described with oriented FAST and rotated BRIEF (ORB) descriptor. Next, it matches the features between the previous frame and the current frame and performs motion-only bundle adjustment that optimizes only the camera pose with fixed map points. The place recognition gets involved in the case of tracking loss. After the camera pose estimation with a group of feature matches, the system projects the created map onto the current frame and looks for further map point correspondences. In case the system detects all the map points in the frame, the system performs a camera pose optimization. For the making decision of keyframe insertion, the tracking thread works with the local mapping thread. Since the local mapping drops the redundant keyframes, the tracking thread attempts to add new keyframes concerning certain conditions. These conditions are listed as follows:

- There should be more than 20 frames processed since the last global relocalization.
- There should be more than 20 frames processed since the latest keyframe addition and local mapping should be idle.
- There should be at least 50 points tracked on the current frame.
- The current frame should track less than 90% points of the reference keyframe.

### 2.4. Cooperative Local Mapping

The local mapper primarily manages the covisibility between the keyframes via the map points and optimizes the local keyframe poses and the map points using local bundle adjustment (LBA). For that purpose, it follows several steps, and these steps are keyframe insertion, map point culling, new map point insertion, local bundle adjustment. and local keyframe culling.

In the processing of a new keyframe, the local mapper updates the covisibility graph and adds a new node for the current keyframe. Then, it links a current keyframe to another keyframe that shares the most map points in common via updating the spanning-tree. Lastly, it calculates a bag of words (BoW) representation (benefits for the data association to triangulate new map points) of the current keyframe.

The local mapper eliminates the low-quality map points repetitively considering two conditions. The first condition is that a tracking thread must see the map point in more than 25% of the frames predicted to be visible. The second one is that at least three keyframes should observe the map point.

The local mapper creates new map points through triangulating ORB from associated keyframes in the covisibility graph. In case there is an unmatched ORB in the current keyframe, the system looks for a match with another unmatched ORB in other keyframes. The ORB match should meet the epipolar constraint to be accepted. To insert a new map point, the local mapper triangulates ORB pairs and checks both frames if they have a positive depth. Additionally, it checks the reprojection error, parallax, and scale consistency.

The LBA optimizes the local keyframes and all the map points observed by the local keyframes. The local keyframes consist of the current keyframe and the other keyframes which are connected to the current keyframe by the covisibility graph [15]. Unlike the original ORB-SLAM2, our modified LBA module optimizes the local keyframes and all the map points observed by the local keyframes and the relative poses between cameras simultaneously. Our LBA module gathers information from all of the cameras and optimizes every map component at once. By this means, the system updates the relative poses between the cameras all the time.

Lastly, to keep a functional simplicity, a local mapper searches for the redundant keyframes and eliminates them from the map. Keyframes, where more than 90% of the map points are observable to three other keyframes with the same scale level, are deleted from the map.

### 2.5. Optimization for an Online Extrinsic Calibration

The proposed system merges the information that comes from multiple cameras to improve the accuracy of the map by an optimization. One possible way is to apply a conventional bundle adjustment which optimizes the map points and all the keyframes registered, even though the keyframes belong to different cameras. However, this requires more degrees of freedom to be optimized than the actual system, because each camera in the rigidly fixed camera system cannot move independently. We utilize the fact that the cameras are rigidly mounted, and thus, the extrinsic parameters must be fixed, while they are not known in advance.

We keep estimating the extrinsic parameters explicitly online by optimizing them along with the other SLAM parameters in the whole sequence. To achieve this, we propose to modify the SLAM formulation in the bundle adjustment. The initial estimates for the extrinsic parameters are obtained by the multi-camera initialization explained in Section 2.2. We convert the SLAM problem of multiple independent cameras into that of the reference camera with additional observations which are observed by the other cameras by using hypotheses of the extrinsic parameters.

**Figure 4:**
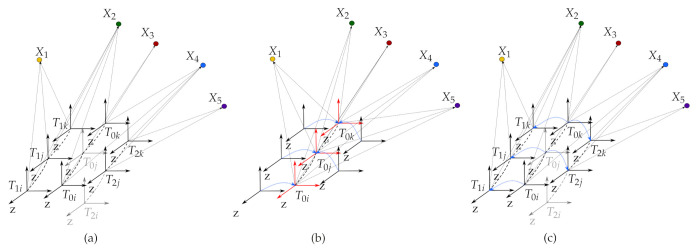
(**a**) The map with the keyframes and the map points before the optimization. Black coordinates represent the keyframes from multi-cameras while gray arrows indicate that there is no keyframe produced at the corresponding timestamp. (**b**) With the help of the extrinsic parameters between the reference camera and the nonreference cameras (blue), all the keyframes are transformed into the corresponding keyframes of the reference camera as if all the map points are observed from the reference camera. (**c**) All the keyframes, the map points, and the extrinsic parameters are optimized. After the optimization, the recovered keyframes of the nonreference cameras are transformed back to the original positions with the help of optimized extrinsic parameters (blue).

To convert the problem, all the keyframes of nonreference cameras are transformed into the corresponding keyframe of the reference camera, as if all the map points are observed from the reference camera. To transform the keyframes of nonreference cameras into those of the reference camera, the system uses the current estimation of the extrinsic parameters. If there are no corresponding keyframes of the reference camera, the system creates sets of *virtual keyframes for the reference camera*. This problem conversion ensures the minimum degree-of-freedom (DOF) parameterization of the unknowns for keyframes to be optimized. Figure 4 presents how the proposed method lowers the degree of freedom to be optimized. As seen in Figure 4a, the number of keyframe parameters to be optimized is 42(= 7∗6DOF), since there are seven keyframes. However, once all the keyframes are transformed into the corresponding keyframe of the reference camera, the number of the keyframe related parameters becomes 30(= 3∗6DOF + 2∗6DOF), where there are three keyframes with two extrinsic parameters between cameras. This difference becomes bigger as the sequence is longer, and the representation with fewer degrees of freedom utilizes more and stronger constraints that limit the solution space. This would help with the stability of the whole system.

We solve the converted problem as a nonlinear minimization and represent a nonlinear cost function describing reprojection errors. A 3D point $X_{p}$ is observed by the camera *c* corresponding reference keyframe *k* whose pose is represented as $T_{0k}$ in the world coordinate, and its observation is given as $x_{kp}^{c}$.

The cost function is to be minimized is the sum of the squared reprojection errors denoted as:

(3) $$\underset{X_{p},T_{0k},T_{0}^{c}}{arg min}\left(\sum_{k,p,c}{x_{kp}^{c}-\rho_{k}\left(X_{p},T_{0k},T_{0}^{c}\right)}^{2}\right).$$

The projection function $\rho_{k}$ is represented as:

(4) $$\rho_{k}\left(X_{p},T_{0k},T_{0}^{c}\right)=\left[\begin{array}{c} f_{ku}\frac{x_{kp}}{z_{kp}}+c_{ku}\\ f_{kv}\frac{y_{kp}}{z_{kp}}+c_{kv} \end{array}\right],$$

(5) $${\left[\begin{array}{c} x_{kp},y_{kp},z_{kp} \end{array}\right]}^{⊤}=R_{k}^{w}X_{p}+t_{k}^{w}$$

where $R_{k}^{w}$ and $t_{k}^{w}$ represent the rotation and translation part of the matrix derived from the following equation:

(6) $$\left[\begin{array}{cc} R_{k}^{w} & t_{k}^{w}\\ 0 & 1 \end{array}\right]=\left[\begin{array}{cc} R_{k0} & t_{k0}\\ 0 & 1 \end{array}\right]\left[\begin{array}{cc} R_{k}^{c} & t_{k}^{c}\\ 0 & 1 \end{array}\right]$$

where $R_{k0}$ and $t_{k0}$ represent the rotation and translation of the reference camera’s keyframe while $R_{k}^{c}$ and $t_{k}^{c}$ represent the rotation and translation of the extrinsic parameters between the reference camera and the nonreference camera, respectively. The focal length $\left(f_{k,u},f_{k,v}\right)$ of the camera with associated principle points $\left(c_{k,u},c_{k,v}\right)$ are known in advance. Note that the relative transforms $T_{0}^{c}$ are also parameters to be optimized as well as the map points $X_{p}$ and the keyframe poses $T_{0k}$.

**Figure 5:**
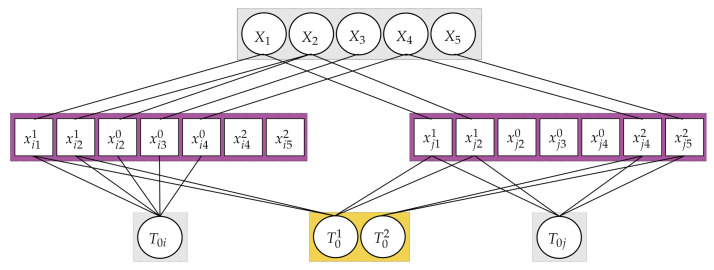
The graph model for the proposed method. Parameters to be optimized and measurements are represented as circles and boxes, respectively. This graph representaton is about Figure 4 at timestamp *i* and *j*. As mentioned, all the nonreference cameras’ keyframes are transformed into the corresponding trajectory of the reference camera. Considering the scenario in Figure 4b, we represent the graph by connecting the reference camera’s keyframes (the gray squares with a white circle), the extrinsic parameters (white circles in the yellow rectangle), 2D map points (white boxes in the purple rectangle), and 3D map points (white set of circles in the gray rectangle). If a map point is observed from a nonreference camera, we use both the corresponding extrinsic parameter and the reference camera’s keyframe for connection to the map points. In case a map point is observed from the reference camera, we then use only the reference camera’s keyframe for connection to the map points.

Like the ORB-SLAM, all optimizations are solved by a graph-based approach in this study. However, in the proposed method, it is not possible to represent the graph by the binary edge that connects two vertices. The main reason is that all the keyframes of nonreference cameras are transformed into the corresponding keyframe of the reference camera. Thus, the extrinsic parameters as unknowns require multi-edges that make connections to two or more vertices, as shown in Figure 5. Even when any two cameras do not see a shared map point, we need to use the multi-edges due to the extrinsic parameters. The representation of the multi-edge based graph-based optimization is shown in Figure 5 for two consecutive timestamps. In this figure, vertices (parameters) are illustrated by circles, and the edges (measurements) are shown as boxes. The scenario presented in Figure 5 is illustrated based on the Figure 4 for timestamp *i* and *j*.

### 2.6. Loop Closing

Each time the system adds a new keyframe, loop closer searches for possible corresponding keyframes in the map to detect a loop. If a loop is detected, the loop closer first calculates a similarity transformation between the current keyframe and the loop candidate keyframe to check the drift in the loop. Then, the system fixes the pose of the current keyframe and covisible keyframes that are connected to the current keyframe to align both sides of the loop using the computed similarity transformation. Next, it merges the duplicated map points by checking the inliers that match both with the current keyframe and the loop keyframe with its neighbors. In the last step of the loop closing, the conventional global bundle adjustment (GBA) fixes all keyframe poses and map points from the initial keyframe whose id is zero. Unlike the original GBA, we use our GBA module based on the multi-edge-based graph optimization to update the extrinsic parameters between cameras, all the keyframes’ poses, and all the map points in the map. Since LBA optimizes the SLAM components locally, the system needs to update everything from scratch using the proposed GBA with the updated extrinsic parameters.

## 3. Experimental Results

To test the performance of the proposed method, various tests are performed on three different datasets created for both indoor and outdoor environments. The first two datasets (EuRoC/ASL and KITTI odometry) are publicly available and set to stereo configuration. The third dataset (KIST) has been created by us that consists of sequences RGB and RGB-D cameras separately. The RGB camera rig consists of three cameras with certain overlaps of their fields of view, and the RGB-D camera rig consists of two cameras that are set similar to stereo configuration. We made comparisons between our method and the other state-of-the-art algorithms on public datasets. Since we use multiple cameras, for the evaluation, we defined the body frame (BF) as the pose of the reference camera.

### 3.1. EuRoC/ASL Dataset

The EuRoC/ASL visual-inertial dataset created by ETH Zurich contains stereo images and synchronized inertial measurement unit (IMU) measurements [17]. The image sequences have been obtained in a small room using a micro aerial vehicle (MAV). Since the proposed method is a visual-based SLAM, we did not use the IMU measurements and use only stereo image sequences independently to test the performance. The sequences used in this experiment are divided into two clusters (V1 and V2) and each cluster includes three scenarios. According to the fast and sharp motions in the small room, these scenarios are named as easy, medium, and difficult. The proposed system is tested on the last six sequences (Vicon Room) of the dataset and compared to the LIBVISO2 [18], S-PTAM [19], LSD-SLAM [20], OKVIS [21,22].

To calculate the absolute trajectory error (ATE), the estimations are aligned to the ground truth using sim(3) Umeyama alignment via the EVO package [23]. Table 2 shows the performance of the proposed method and the comparison with the other state-of-the-art algorithms in terms of the RMSE error of the estimated trajectories. The best results are shown in *bold*, and the second-best results are indicated in *blue*. The results show that the proposed method achieves the best accuracy in all sequences.

**Figure 6:**
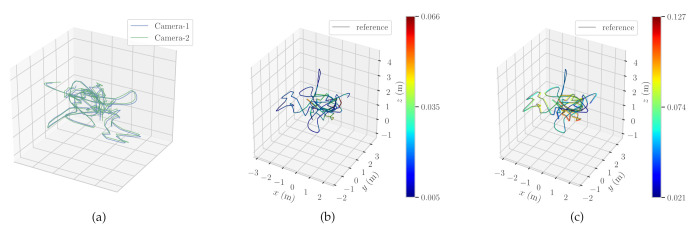
(**a**) The reference and nonreference camera trajectories in EuRoc/ASL V1_03 sequence. (**b**,**c**) The comparison of the left and the right camera with the ground truth data on EuRoc/ASL V1_03. The color bar represents the error of the estimated trajectory compared to the ground truth data. As the trajectory turns into blue, the error gets smaller.

Besides, the experiments also confirm that the relative poses between cameras stay stable throughout the entire trajectories of the dataset. Figure 6a shows how the relative pose between the left and the right cameras remains stable throughout the *sequence-V1_03*. To show the performance of the trajectory estimation, the comparison of the proposed method with the ground truth data is presented in Figure 6b,c on the *sequence-V1_03*.

### 3.2. KITTI-Odometry Dataset

The KITTI odometry dataset [24] includes two different camera pairs that are set in a stereo configuration. The proposed system is tested on the first eleven sequences of the dataset and compared to the LIBVISO2 [18], S-PTAM [19,25]. To show the effect of the refinement of the map components, the proposed system is also compared to itself without using the proposed optimization. Instead, the conventional ORB-SLAM2’s optimization module is used to optimize the keyframe poses and the map points. For the calculation of the absolute trajectory error (ATE), the estimations are aligned to the ground truth using sim(3) Umeyama alignment via the EVO package [23]. Table 3 shows the comparison between the proposed method and the other state-of-the-art algorithms in terms of the RMSE error of the estimated trajectories. The best results are shown in *bold*, and the second-best results are indicated in *blue*. The proposed system has the best performance on the *sequence 00, 02, 03* while it has comparable performances for the rest of the sequences. Additionally, Table 3 shows the necessity of the refinement since the proposed optimization has better performance.

**Figure 7:**
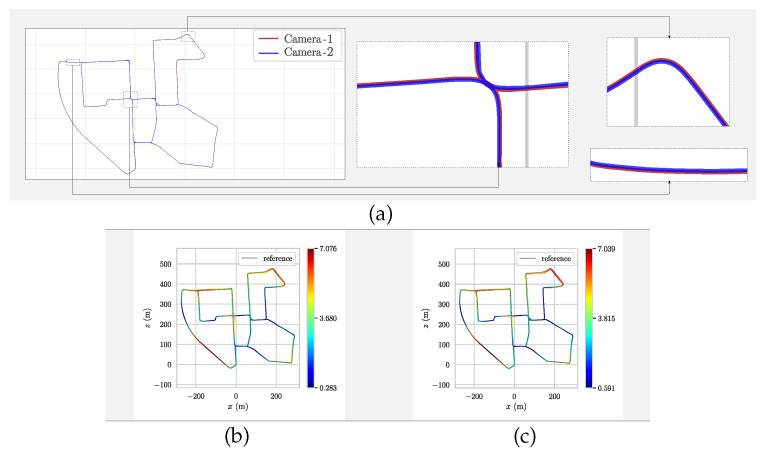
(**a**) The multi-camera trajectories using the proposed method in KITTI-00. (**b**,**c**) The comparison of the left and the right camera with the ground truth data on KITTI-00. The color bar represents the error of the estimated trajectory compared to the ground truth data. As the trajectory turns into blue, the error gets smaller.

All of the state-of-the-art algorithms in this comparison are precalibrated based stereo SLAM systems. Considering this, our proposed method shows comparable results, even though there are no given extrinsic parameters in advance. Furthermore, the average ATE of the trajectory estimations shows that the proposed method has improved the performance of the conventional ORB-SLAM2. Another result showing how accurate the proposed method is that the relative poses between cameras remain stable throughout the entire trajectories of the dataset. Figure 7a shows how the relative pose between the left and the right cameras remains constant in the *sequence 00*. Figure 7b also shows the performance of each camera compared to the ground truth. As one can notice, the proposed method showed a better performance for the EuRoC/ASL than for the KITTI dataset. It would be caused by the motion characteristics: the KITTI dataset is captured by cameras fixed on a vehicle, and their movement is limited only in a plane, but the EuRoC/ASL dataset is captured by cameras on a micro aerial vehicle which moves freely in 6 DOF.

### 3.3. KIST-Dataset

The last dataset used in this experiment has been made by the department of the Center for Intelligent and Interactive Robotics, Korea Institute of Science and Technology (KIST). The dataset has sequences captured by RGB and RGB-D cameras.

For the RGB camera data, the camera rig consists of three separate cameras with small overlapping regions. The images have 1288 × 964 resolutions and were captured in 30 fps by using FLIR Chameleon3 cameras. The dataset consists of three sequences that contain loops and is a composition of image sequences captured from an indoor environment. All the images between cameras are synchronized properly. The first sequence (KIST-00) is taken in a large hall that is 550 m${}^{2}$. The second sequence (KIST-01) consists of a set of images taken in a small laboratory that is 78.8 m${}^{2}$. The last RGB sequence (KIST-02) is taken in a long narrow set of corridors with 9×6 m lengths.

**Figure 8:**
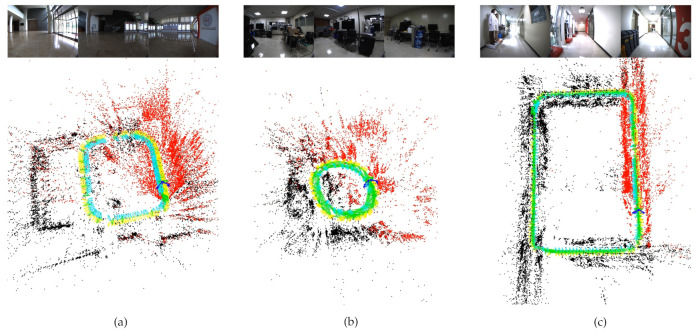
The multi-camera trajectories of the proposed method with some real image sequences of the scenarios. (**a**) KIST-00, (**b**) KIST-01, and (**c**) KIST-02 sequences. The current reference camera is indicated green while the nonreference cameras are shown with navy color. Additionally, each camera’s keyframes are shown with different colors. The keyframes created by the reference camera are shown with green color, and the nonreference cameras’ keyframes are indicated yellow and turquoise colors. Additionally, local map points are shown with red color, while black map points represent the fixed map points that are not being processed currently.

Unlike the EuRoC/ASL and the KITTI datasets, the cameras are outward-looking and not a stereo camera, and it provides a much smaller overlapping region in the dataset. Even though the overlapping region is small, relative poses between cameras stay stable for whole sequences. Figure 8 shows the entire trajectories of RGB data sequences.

**Figure 9:**
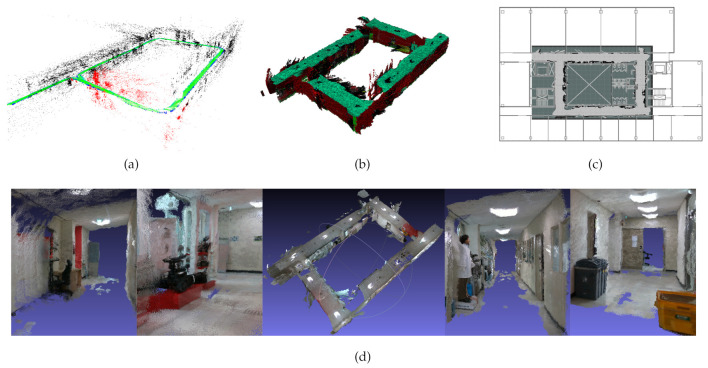
Different forms of the output of the KIST-03 sequence using RGB-D sensors. (**a**) sparse map of the environment, (**b**) 3D octomap of the map, (**c**) 2d occupancy gridmap aligned with the floor plan, (**d**) RGB pointcloud of the environment.

For the RGB-D camera data, a sequence (KIST-03) is taken using two Intel Realsense D435 RGB-D sensors (640 × 480 resolutions with 30 fps) in the same environment with KIST-02. In this sequence, we used unsynchronized camera sequences. To evaluate the performance of the system, we first created a 3D octomap with a ROS package [26]. Next, we extracted the 2D occupancy grid map concerning the created 3D octomap. We then align the 2D occupancy grid map on the building’s floorplan. The output of the RGB-D experiment are listed as follows: the sparse map of an environment (Figure 9a), 3D octomap (Figure 9b), 2D occupancy gridmap on the floor plan (Figure 9c), and the pointcloud of the environment from different viewpoints (Figure 9d). According to Figure 9c, it is possible to say that the scale is consistent and shows how the map points are accurate using the proposed system.

## 4. Conclusions

In this study, we have introduced a novel approach for a multi-camera SLAM system, which does not require precalculated extrinsic parameters between cameras. We have aimed to merge the information that comes from independently working multiple cameras to improve the accuracy of the map by an optimization. Unlike a conventional multi-camera SLAM system which regards all the cameras as a single unit, we have proposed to track each camera independently while a shared map maintains for all cameras. By doing that, the tracking failure caused by erroneous extrinsic parameters is avoided. To improve the map accuracy, we have also proposed a new bundle adjustment which keeps refining the extrinsic parameters online as well as the map points and the keyframe poses. We have converted from a bundle adjustment for conventional multiple cameras into that for multiple cameras fixed to each other to obtain the minimum number of unknowns to be optimized. A new initialization method has been introduced for the map and multiple uncalibrated cameras on the single map. To test the performance of our method, we have used three different datasets that include 21 sequences. The experiments have shown that our system works robustly and accurately with various camera configurations in both indoor and outdoor environments. The fact that our system gives accurate results in scenarios with different numbers of cameras and different viewing angles proves the consistency of the proposed method, while our method does not require a precalibration of the multi-camera system at all.

## Figures and Tables

**Table 1:** Definitions of the SLAM components.

Map Components	Definition
$X_{p}$	The 3D map point, where *p* is map point index.
$x_{kp}^{c}$	The 2D projection of the map point, where *k* is the keyframe index, *p* is the map point index and *c* is the camera index.
$T_{ck}$	The pose of keyframes, where *c* is the camera index and *k* is the keyframe index.
$T_{i}^{j}$	The relative poses between cameras *i* and *j*, or the extrinsic parameters.

**Table 2:** Comparison of root mean square error (RMSE) in meters on EuRoC/ASL dataset. The median values are shown with *(m)*. The best results are shown in *bold*, and the second-best results are indicated in *blue*.

	Proposed Method	[ORB-SLAM2]	[LIBVISO2 *]	[S-PTAM *]	[LSD-SLAM *]	[OKVIS *]	
Sequence	Body Frame (BF)	Monocular	Stereo	Stereo	Stereo	Stereo	Stereo
V1_01 (easy)	**0.008** (m: 0.006)	0.088	0.26	0.20	0.19	0.08	0.11
V1_02 (medium)	**0.046** (m: 0.042)	0.065	0.17	0.58	0.58	0.61	0.14
V1_03 (difficult)	**0.023** (m: 0.021)	0.091	0.24	-	-	0.14	0.36
V2_01 (easy)	**0.029** (m: 0.019)	0.066	0.53	1.88	0.45	0.10	0.11
V2_02 (medium)	**0.036** (m: 0.035)	0.062	0.92	-	0.51	0.18	0.27
V2_03 (difficult)	**0.055** (m: 0.042)	-	-	-	-	0.24	-
	* numbers are taken from [21].			

**Table 3:** Comparison of root mean square error (RMSE) in meters on KITTI dataset. The median values are shown with (m). The best results are shown in *bold*, and the second-best results are indicated in *blue*.

	Proposed Method	Proposed Method	ORB-SLAM2	LIBVISO2 *	S-PTAM *	
Sequence	BF (with Refinement)	BF (w/o Refinement)	Monocular	Stereo	Stereo	Stereo
00	**3.66** (m: 2.75)	3.72	5.33	29.71	7.83	5.79
01	-	-	-	66.54	204.65	**61.55**
02	**17.62** (m: 12.94)	20.15	21.25	34.26	20.78	18.99
03	**0.58** (m: 0.51)	1.01	1.51	1.67	10.53	0.63
04	1.33 (m: 1.10)	1.29	1.62	0.80	0.98	**0.67**
05	4.03 (m: 3.85)	4.54	4.85	22.14	**2.80**	5.47
06	11.49 (m: 11.59)	13.74	12.34	11.54	4.00	**2.06**
07	2.11 (m: 1.84)	2.22	2.26	4.41	**1.80**	2.34
08	33.42 (m: 21.65)	47.98	46.68	47.67	**5.13**	8.42
09	6.65 (m: 5.40)	6.81	6.62	89.83	7.27	**5.46**
10	6.72 (m: 3.67)	6.79	8.80	49.35	2.08	**1.68**
	* numbers are taken from [25].		

## Data Availability

Data available in a publicly accessible repository. First two dataset presented in this study are openly available in [17,24]. For the third dataset, data sharing is not applicable.
